# B1-based SAR reconstruction using contrast source inversion–electric properties tomography (CSI-EPT)

**DOI:** 10.1007/s11517-016-1497-6

**Published:** 2016-04-23

**Authors:** Edmond Balidemaj, Cornelis A. T. van den Berg, Astrid L. H. M. W. van Lier, Aart J. Nederveen, Lukas J. A. Stalpers, Hans Crezee, Rob F. Remis

**Affiliations:** 1 Radiotherapy Department, Academic Medical Center, University of Amsterdam, Amsterdam, The Netherlands; 2 Radiotherapy Department, University Medical Center, Utrecht University, Utrecht, The Netherlands; 3 Radiology Department, Academic Medical Center, University of Amsterdam, Amsterdam, The Netherlands; 4 Circuits and Systems Group, Faculty of Electrical Engineering, Mathematics and Computer Science, Delft University of Technology, Delft, The Netherlands

**Keywords:** B1 maps, Specific absorption rate (SAR), Contrast source inversion (CSI), Electric properties tomography (EPT), Conductivity

## Abstract

Specific absorption rate (SAR) assessment is essential for safety purposes during MR acquisition. Online SAR assessment is not trivial and requires, in addition, knowledge of the electric tissue properties and the electric fields in the human anatomy. In this study, the potential of the recently developed CSI-EPT method to reconstruct SAR distributions is investigated. This method is based on integral representations for the electromagnetic field and attempts to reconstruct the tissue parameters and the electric field strength based on $B_{1}^{ + }$ field data only. Full three-dimensional FDTD simulations using a female pelvis model are used to validate two-dimensional CSI reconstruction results in the central transverse plane of a 3T body coil. Numerical experiments demonstrate that the reconstructed SAR distributions are in good agreement with the SAR distributions as determined via 3D FDTD simulations and show that these distributions can be computed very efficiently in the central transverse plane of a body coil with the two-dimensional approach of CSI-EPT.

## Introduction

Assessment of the specific absorption rate (SAR) due to electromagnetic (EM) fields in human tissue is relevant in many applications such as hyperthermia [9, 10, 16], telecommunications [23], and high field MRI [5, 8, 21, 30, 35]. However, for reliable SAR assessment, knowledge of the electric properties (EPs) of biological tissues is required (in particular, the conductivity $\sigma$ and permittivity $\varepsilon$) and the electric field strength must be known as well. This information is usually not directly available and therefore has to be determined by other means. In MRI, various implementations of electric properties tomography (EPT) methods have been developed to extract this information from the $B_{1}^{ + }$ field [1, 12, 13, 19, 24, 33, 34, 36]. This field is accessible to measurement and present-day EPT methods attempt to reconstruct the electric tissue parameters from measured $B_{1}^{ + }$ field maps, while the corresponding electric field strength is determined by forward modeling in which the reconstructed conductivity and permittivity profiles serve as a model for the patient’s anatomy.

One of the drawbacks of the EPT methods mentioned above is that these methods typically suffer from reconstruction artifacts especially near tissue boundaries. These artifacts occur mainly because currently used EPT methods are based on local field equations (either Maxwell’s equations or Helmholtz’s equation) and do not take the electromagnetic boundary conditions into account. Furthermore, these methods are very sensitive to noise or other perturbations in the data, since differential operators act on measured $B_{1}^{ + }$ field data. Different studies have focused on minimizing the reconstruction artifacts by using either the gradient of EP profiles in conjunction with a multi-channel transmit/receive array RF coil [20] or arbitrary-shaped kernels based on voxel position [15]. However, these ad hoc solutions are still based on a local differential operator approach, which may yield less accurate SAR predictions due to potential reconstruction errors in the EP profiles that immediately affect the computed electric field strength in the forward modeling step.

As an alternative to local EPT methods, we have recently proposed an iterative contrast source inversion EPT method (CSI-EPT) [2], which is based on global integral representations for the electromagnetic field [2, 3]. The electromagnetic boundary conditions are then automatically satisfied and reconstruction results near tissue interfaces are significantly improved [2]. Furthermore, CSI-EPT is less sensitive to noise since in CSI-EPT integral operators act on measured field data (instead of differential operators as in local EPT methods) and CSI-EPT reconstructs the electric field strength inside the region of interest as well. This latter property makes CSI-EPT an ideal candidate for SAR reconstructions based on $B_{1}^{ + }$ field data, since it attempts to simultaneously reconstruct the EP profiles and the electric field strength within the human anatomy.

The electromagnetic wave field inside the human body is obviously a fully vectorial three-dimensional wave field. However, as earlier described by van de Bergen [31], the electromagnetic field in the central transverse plane of a 3T or 7T body coil can be treated as a two-dimensional wave field where only $H_{x}$, $H_{y}$, and $E_{z}$, are present. The case where only $H_{x}$, $H_{y}$, and $E_{z}$ are considered is also referred to as the TM-polarized case. Reconstructing the SAR distribution based on two-dimensional instead of three-dimensional fields obviously leads to significant speedups in computation time and may even allow for online SAR reconstructions. Our approach is therefore to reconstruct the SAR distribution in the neighborhood of the central transverse plane of a body coil using a two-dimensional CSI-EPT reconstruction method. To validate our approach, we compare the reconstructed profiles, electric fields, and SAR distributions with 3D models and fully vectorial 3D FDTD simulations. We use a static field of 3T in all numerical experiments. The approach is equally applicable for 7T or other static background field strengths, as long as the two-dimensional field approximation in the central slice remains valid.

## Methods

### The CSI-EPT method

In this section, we briefly discuss the main features of the CSI-EPT method. The method is fully described in [2] and further mathematical details can be found in [27] and [28].

As a starting point, we first write the RF field {*E*, $B_{1}^{ + }$} that is present in the MR system as a superposition of the electromagnetic background field and the scattered field. The background field {$E^{\text{b}}$, $B_{1}^{{ + ;{\text{b}}}}$} is the field that is present within the MR system in the absence of a dielectric object or body, whereas the scattered field {$E^{\text{sc}}$, $B_{1}^{{ + ;{\text{sc}}}}$} is the field induced by the object or body. The object occupies a bounded domain $D$ and is characterized by a conductivity $\sigma \left( r \right)$, a permittivity $\varepsilon \left( r \right)$, and a permeability $\mu \left( r \right)$, with $r = \left( {x,y,z} \right)$ the position vector. In this work, we have ignored relative permeability variations as they are considered negligible for biological tissue [7]. In practice, the background field {$E^{b}$, $B_{1}^{{ + ;{\text{b}}}}$} can be acquired by forward modeling.

Using the linearity of Maxwell’s equations, the scattered electric field at a point with position vector *r* can be written as [27]

1 $$E^{\text{sc}} \left( r \right) = \mathop \int \limits_{{r^{{\prime }} \in D}}^{{}} G^{EJ} \left( {r,r^{{\prime }} } \right)w\left( {r^{{\prime }} } \right){\text{d}}V,$$

while the scattered $B_{1}^{{ + ;{\text{sc}}}}$ can be written as

2 $$B_{1}^{{ + ;{\text{sc}}}} \left( r \right) = \mathop \int \limits_{{r^{{\prime }} \in D}}^{{}} G^{ + ;HJ} \left( {r,r^{{\prime }} } \right)w\left( {r^{{\prime }} } \right){\text{d}}V.$$

In these equations, $G^{EJ}$ denotes the Green’s tensor relating the electric current to electric field and the tensor $G^{ + ;HJ}$ relates the electric current to the $B_{1}^{ + }$ field. Furthermore, $w$ is the contrast source given by

3 $$w = \chi E,$$

where $\chi = \eta /\eta_{b} - 1$ is the contrast function, with $\eta \left( r \right) = \sigma \left( r \right) - i\omega \varepsilon \left( r \right)$, and $\eta_{b} = - i\omega \varepsilon_{0}$.

Obviously, the goal is to reconstruct the contrast function $\chi$ and the electric field $E$ based on $B_{1}^{ + }$ data. A solution to this inverse problem is formulated by iteratively minimizing the cost function given by

4 $$F = F_{\text{data}} + F_{\text{obj}}$$

where

5 $$F_{\text{data}}^{\left[ n \right]} = \frac{{B_{1}^{{ + ;{\text{sc}}}} - \hat{G}^{ + ;HJ} \left\{ {w^{\left[ n \right]} } \right\}}}{{B_{1}^{{ + ;{\text{sc}}}} }}$$

and

6 $$F_{\text{obj}}^{\left[ n \right]} = \frac{{\chi^{\left[ n \right]} E^{\left[ n \right]} - w^{\left[ n \right]} }}{{\chi^{{\left[ {n - 1} \right]}} E^{b} }}$$

where we have introduced the operator $\hat{G}^{ + ;HJ} \left\{ w \right\}$ as

7 $$\hat{G}^{ + ;HJ} \left\{ w \right\}\left( r \right) = \mathop \int \limits_{{r^{{\prime }} \in D}}^{{}} G^{ + ;HJ} \left( {r,r^{{\prime }} } \right)w\left( {r^{{\prime }} } \right){\text{d}}V.$$

The subscript $\left[ n \right]$ in (5) and (6) represents the iteration number. The CSI method updates both the contrast source ($w^{\left[ n \right]}$) and the contrast function ($\chi^{\left[ n \right]}$) using a two-step updating procedure. In the first step, the contrast function is fixed ($\chi = \chi^{{\left[ {n - 1} \right]}}$) while the contrast source ($w^{\left[ n \right]}$) is updated by minimizing Eq. (5). In the second step, a new contrast function ($\chi^{\left[ n \right]}$) is obtained by using the updated contrast source $w^{\left[ n \right]}$ from the first step. Moreover, the electric field corresponding to the updated $w^{\left[ n \right]}$ can be computed by

8 $$E^{\left[ n \right]} = E^{\text{b}} \left( r \right) + \hat{G}^{EJ} \left\{ {w^{\left[ n \right]} } \right\}$$

with

9 $$\hat{G}^{EJ} \left\{ w \right\}\left( r \right) = \mathop \int \limits_{{r^{\prime} \in D}}^{{}} G^{EJ} \left( {r,r^{{\prime }} } \right)w\left( {r^{{\prime }} } \right){\text{d}}V.$$

Finally, the contrast function is then obtained by minimizing Eq. (6) with respect to $\chi$; hence, the new contrast function is computed as

10 $$\chi^{\left[ n \right]} = \frac{{w^{\left[ n \right]} \bar{E}^{\left[ n \right]} }}{{E^{\left[ n \right]} \bar{E}^{\left[ n \right]} }}.$$

The overbar in Eq. (10) denotes the complex conjugate. The iterative process is terminated once the cost function, Eq. (4), reaches a user-specified tolerance level. Elsewhere we reported a more detailed description of the CSI-EPT algorithm [2] which includes the multiplicative total variation factor for noise suppression and the ability to include more than one B1 data set in the iterative process.

### 3D and 2D electromagnetic modeling

We have performed 3D field simulations using in-house developed finite-difference time domain (FDTD) tools [25] and the 3T body coil model as described in [29]. The coil was tuned at 128 MHz (i.e., the Larmor frequency at 3T) and was driven in quadrature mode. The female body model (Ella) from the virtual family provided by IT’IS [6] has been used, and the assigned conductivity and permittivity values are based on [11] at 128 MHz. The tissue density values reported in [14] were used for SAR computations. The computed SAR by 3D field simulations serves as a benchmark to which the 2D simulations will be compared.

The 2D simulations (for a TM-polarized configuration) were conducted using the integral equation method. In the TM-polarized configuration, the electric field vector is parallel to the invariance direction. The fields were generated by 16 RF line sources driven at 128 MHz, which corresponds to an operating frequency of the RF body coil in a 3T MR system. The line sources were located on a circle ($R = 0.34$ m) symmetrically positioned around the female pelvis model with an isotropic voxel size of 2.5 mm. A homogeneous medium (free space) is taken as a background model. In the current implementation, we have assumed exact knowledge of the $B_{1}^{ + }$ phase.

The CSI-EPT algorithm is implemented as we previously described in [2]. The CSI-EPT software code was implemented in MATLAB (MathWorks, Natick, Massachusetts, USA). The computational time for 5000 iterations of the presented method, with a grid size of 2.5 mm, is around 110 s on an Intel Core i7 operating at 1.9 GHz. Furthermore, SAR_1g_ and SAR_10g_, representing the average SAR over a mass of 1 and 10 g, respectively, are computed based on [4] and take approximately 20 and 10 s, respectively.

We have compared the results for the mid-plane slice ($z = 0$ cm) as the 2D modeling is likely to be a valid approximation in this region. However, we have also explored the reconstruction at two off-central slices (i.e., $z = + 7.5$ cm and $z = - 2.5$ cm).

## Results

**Fig. 1:**
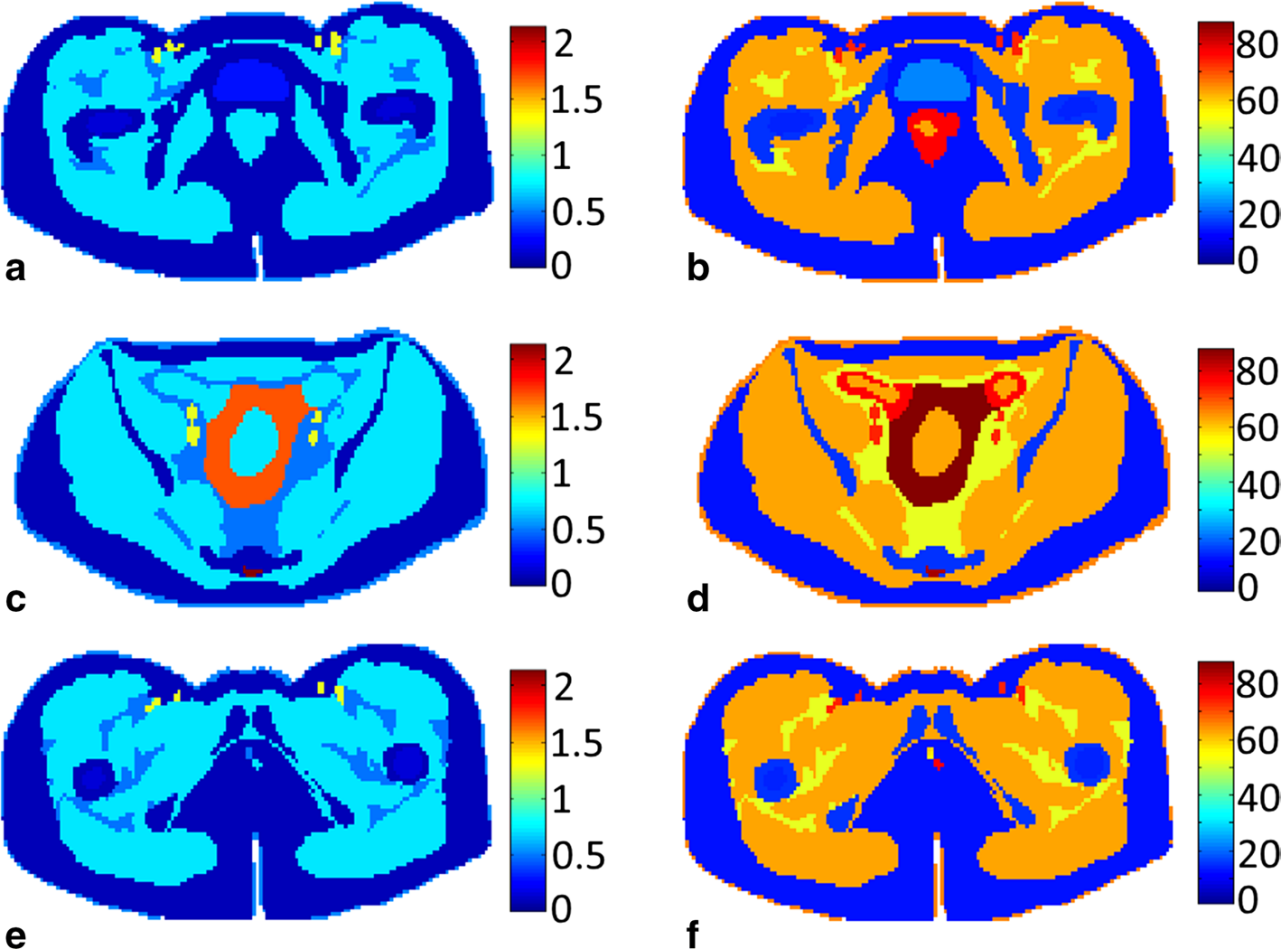
Target electric conductivity (*left*) and permittivity maps (*right*) of the midplane slice (*top row*), the slice at $z = + 7.5$ cm (*middle row*) and the slice at $z = - 2.5$ cm (*bottom row*)

**Fig. 2:**
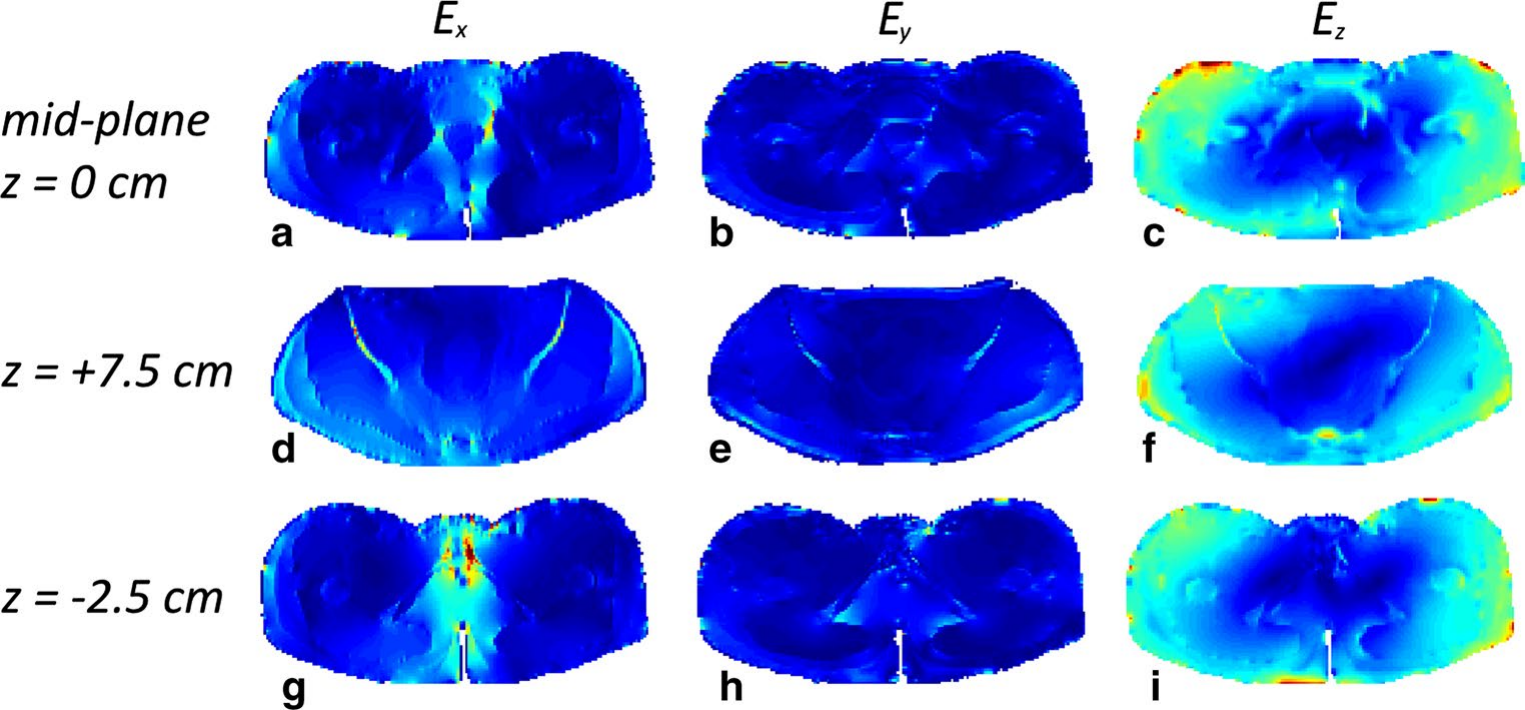
$\left| {E_{x} } \right|,\left| {E_{y} } \right|, {\text{and}} \left| {E_{z} } \right|$ (*left* to *right*) distributions in the midplane slice (*top row*), the slice at $z = + 7.5$ cm (*middle row*), and the slice at $z = - 2.5$ cm (*bottom row*). The $\left| {E_{x} } \right|,\left| {E_{y} } \right|$ field distributions are normalized with respect to the maximum amplitude of the corresponding $\left| {E_{z} } \right|$ field distribution

**Fig. 3:**
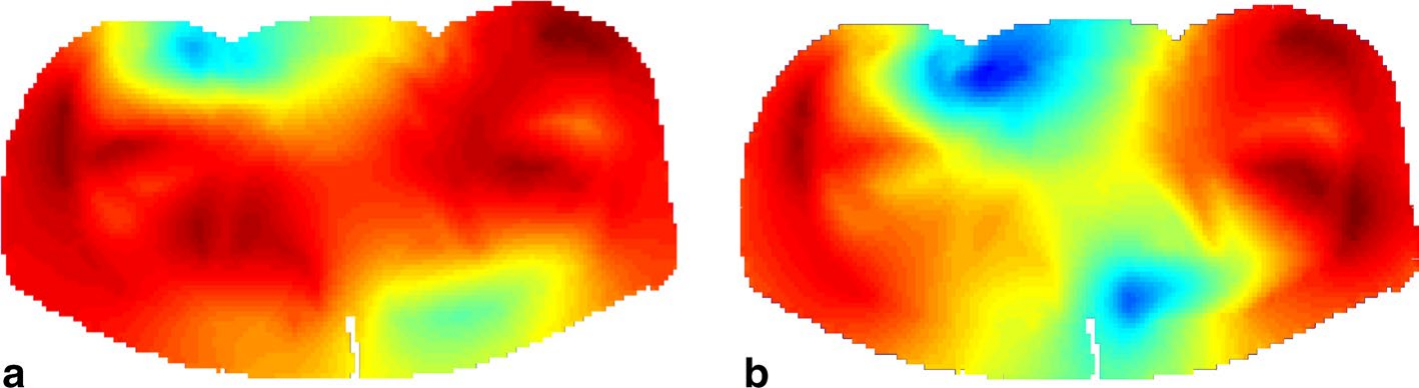
Normalized $\left| {B_{1}^{ + } } \right|$ field distribution in the midplane slice based on 3D FDTD (**a**) and the 2D integral equation method (**b**)

To test the SAR reconstruction results of our algorithm, we first compute the fully three-dimensional electromagnetic field inside the 3D Ella body model using FDTD and focus on the field and SAR distributions in three slices located at $z = 0$ cm (midplane), $z = + 7.5$ cm, and $z = - 2.5$ cm. The conductivity and permittivity profiles within these three slices are shown in Fig. 1, while the magnitude of the Cartesian components of the corresponding 3D electric field strength is shown in Fig. 2. In these figures, the amplitudes of the field components $E_{x}$ and $E_{y}$ are normalized with respect to the maximum amplitude of the $E_{z}$ field of the corresponding slice. We observe that $E_{z}$ is the dominant field component in all three slices indicating that it is reasonable to assume a two-dimensional E-polarized field structure in and around the midplane of the body coil. The field is not exactly two-dimensional, of course, which is particularly noticeable for the *x*-component of the electric field strength (first column of Fig. 2). This component vanishes for a two-dimensional E-polarized field, but it clearly does not in the fully three-dimensional case especially around the center of the slices and within the slice located closed to the legs (slice at $z = - 2.5$ cm). These deviations from 2D are due to anatomical variations in the longitudinal *z*-direction, which are especially large around the slice located at $z = - 2.5$ cm, since here we transited from the torso to the upper legs. Finally, the 3D- and 2D-normalized $\left| {B_{1}^{ + } } \right|$ maps of the midplane slice are shown in Fig. 3a, b, respectively. We observe that both maps have a similar field pattern, apart from some local differences mainly at the central region. This observation again confirms that it is reasonable to assume that the electromagnetic field essentially has a two-dimensional E-polarized field structure in the midplane of the body coil.

**Fig. 4:**
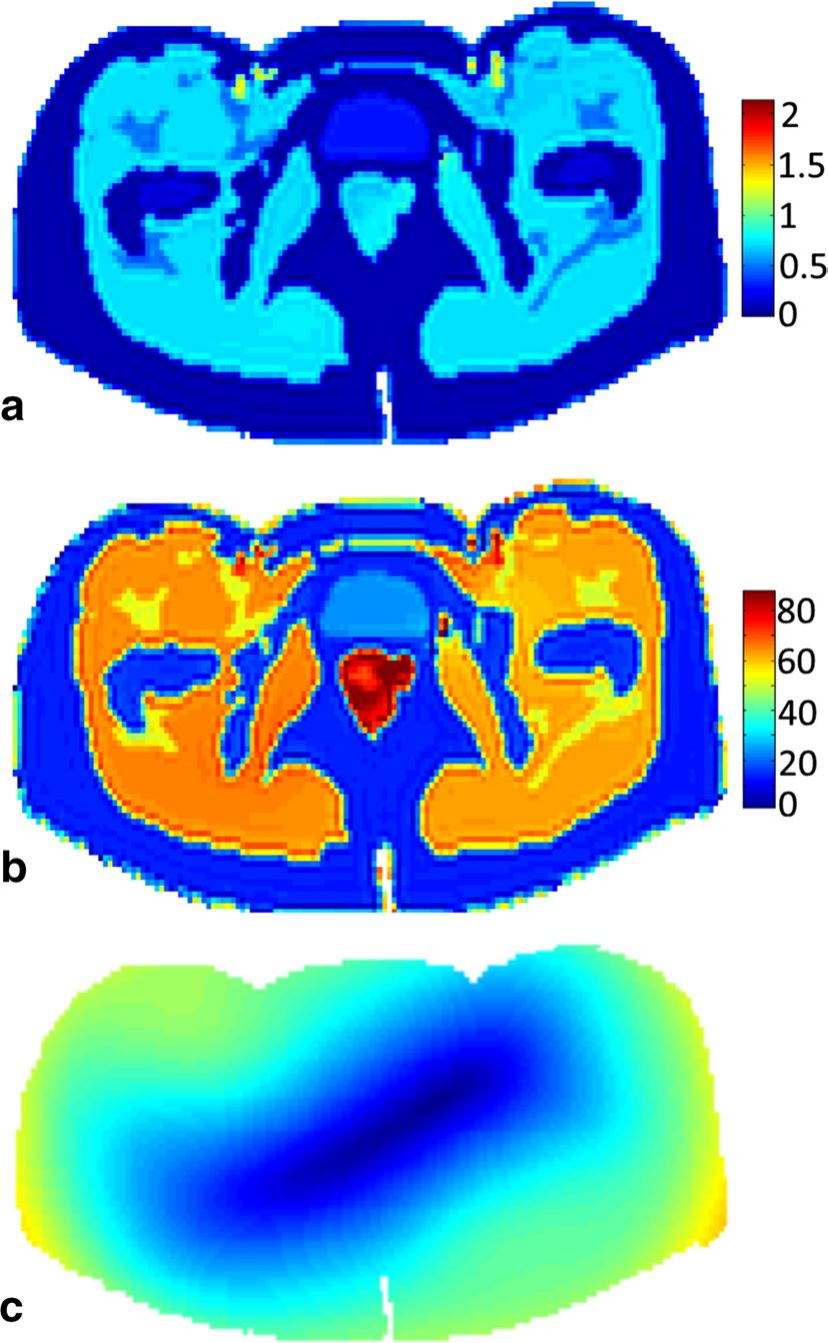
The reconstructed conductivity (**a**) and permittivity (**b**) maps after 5000 iterations of the CSI-EPT algorithm. **c** The normalized $\left| {E_{z} } \right|$

In practice, the measured $B_{1}^{ + }$ field is not known exactly, of course, and we therefore contaminate the 2D-simulated $B_{1}^{ + }$ field with additive Gaussian noise (SNR 20). This field now serves as an input for our CSI-EPT algorithm. The reconstructed conductivity and permittivity maps obtained after 5000 iterations of the CSI-EPT algorithm are shown in Fig. 4a, b, respectively. We note that these results were obtained by incorporating multiplicative total variation regularization into our CSI-EPT algorithm (for details, see [2]) to suppress the effects of noise in the data. From Fig. 4a, b, we observe that the conductivity and permittivity reconstructions are in good agreement with the target maps of Fig. 1a, b. Furthermore, in Fig. 4c, the reconstructed $\left| {E_{z} } \right|$ field is shown which is used together with the reconstructed conductivity map of Fig. 4a to determine the SAR distribution as reconstructed by CSI-EPT.

**Fig. 5:**
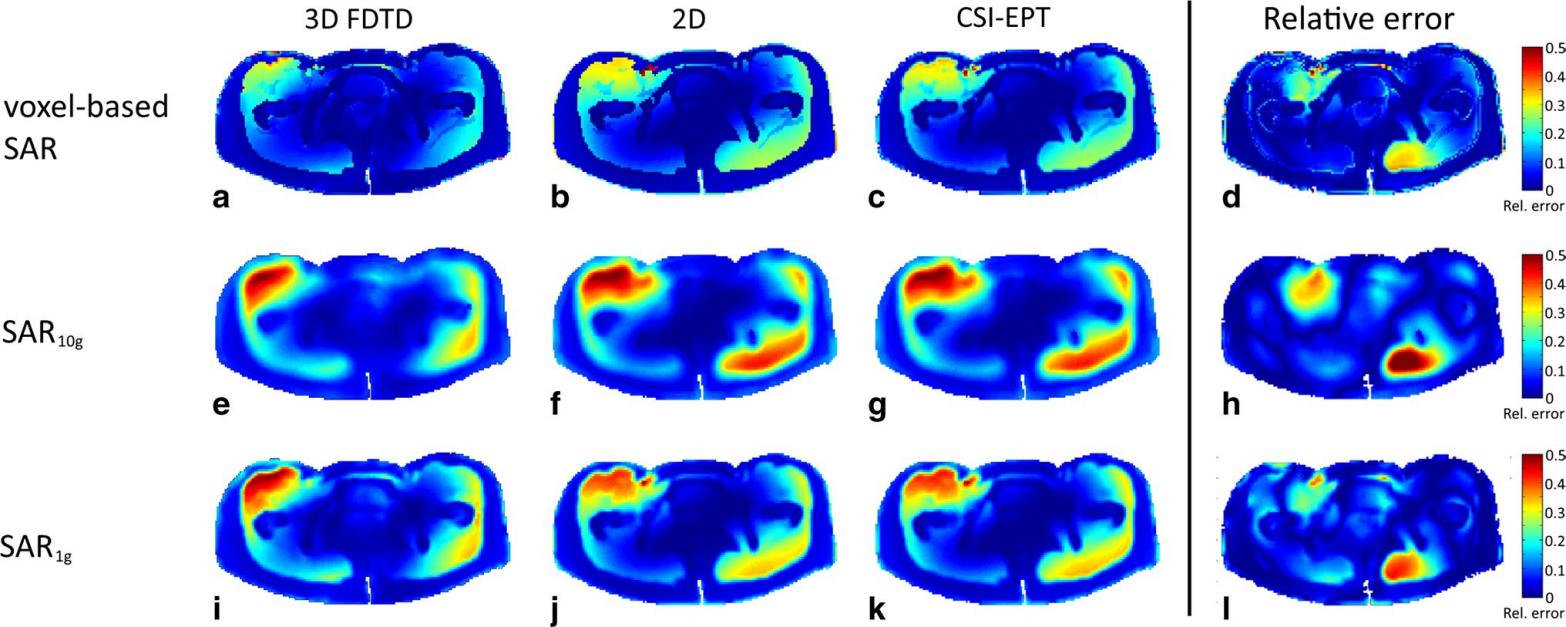
The normalized voxel-based SAR distribution (*top row*), the normalized SAR_10g_ distribution (*middle row*), and the SAR_1g_ distribution (*bottom row*) of the midplane slice ($z = 0$  cm). The distributions based on 3D FDTD field simulations are shown in (**a**, **e**, **i**), while the distributions based on a 2D integral equation approach are shown in (**b**, **f**, **j**). The reconstructed SAR distributions based on CSI-EPT are presented in (**c**, **g**, **k**). The relative error between the reconstructed SAR distributions based on CSI-EPT and 3D FDTD is shown in (**d**, **h**, **l**)

Figure 5a shows the voxel-wise SAR distribution based on 3D field simulations, while the SAR_10g_ and SAR_1g_ are depicted in Fig. 5d, g, respectively. In Fig. 5b, e, h (second column of Fig. 5), the computed SAR distributions based on the 2D field simulations are shown, which are in good agreement with the distributions based on the 3D simulations (1st column of Fig. 5). Only slight deviations are observed on the right bottom part of the anatomy. Finally, the CSI-SAR reconstructions using only $B_{1}^{ + }$ field information are shown in Fig. 5c, f, i (third column of Fig. 5). As mentioned above, this $B_{1}^{ + }$ field is contaminated with additive Gaussian noise (SNR 20). Comparing the different reconstructed SAR distributions with the 3D (first column of Fig. 5) and 2D (second column of Fig. 5) SAR distributions, we observe that the CSI-SAR reconstructions are in good agreement with the 3D- and 2D-modeled SAR distributions. The relative error between the reconstructed SAR distributions based on CSI-EPT and 3D FDTD is shown in the fourth column of Fig. 5.

**Fig. 6:**
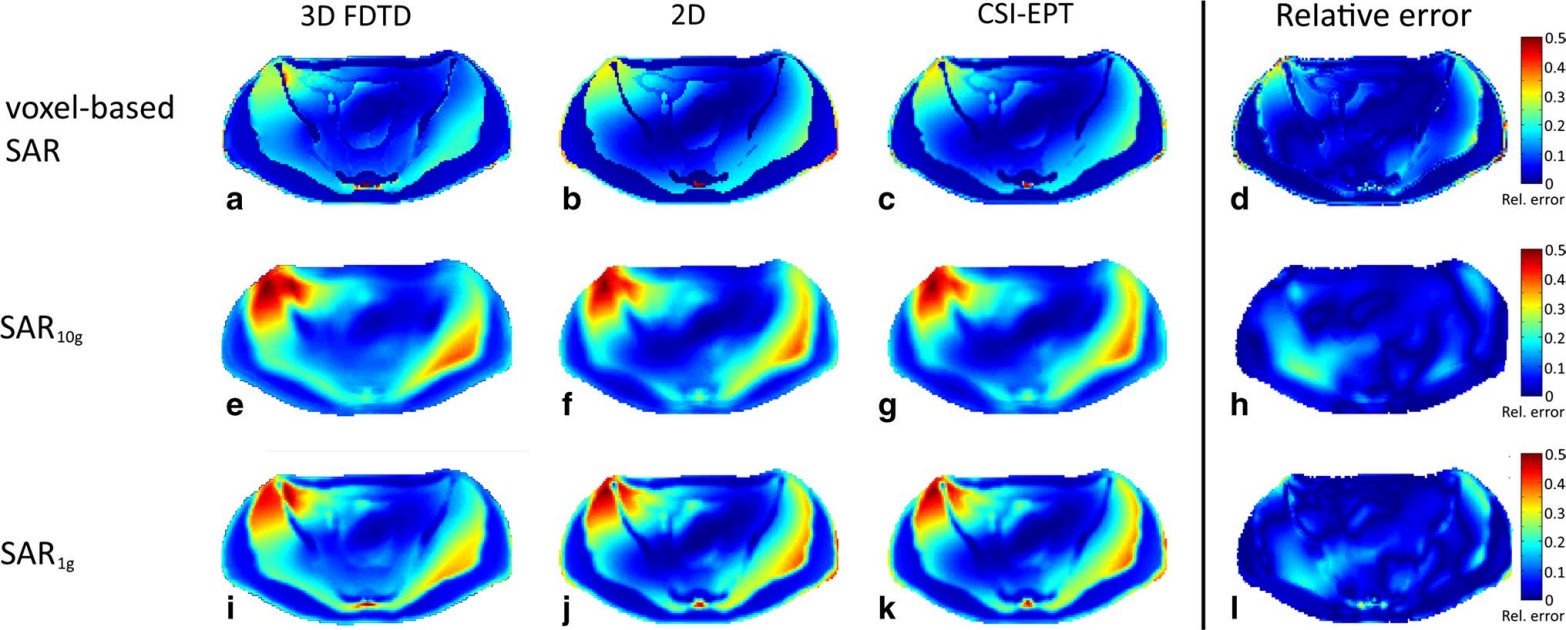
The normalized voxel-based SAR distribution (*top row*), the normalized SAR_10g_ distribution (*middle row*), and the SAR_1g_ distribution (*bottom row*) of the slice at $z = + 7.5$  cm. The distributions based on 3D FDTD field simulations are shown in (**a**, **e**, **i**), while the distributions based on a 2D integral equation approach are shown in (**b**, **f**, **j**). The reconstructed SAR distributions based on CSI-EPT are presented in (**c**, **g**, **k**). The relative error between the reconstructed SAR distributions based on CSI-EPT and 3D FDTD is shown in (**d**, **h**, **l**)

**Fig. 7:**
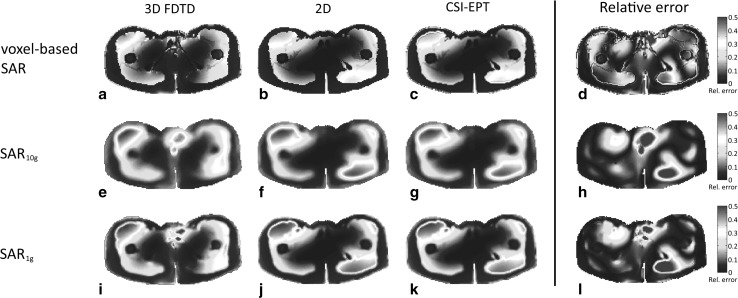
The normalized voxel-based SAR distribution (*top row*), the normalized SAR_10g_ distribution (*middle row*), and the SAR_1g_ distribution (*bottom row*) of the slice at $z = - 2.5$  cm. The distributions based on 3D FDTD field simulations are shown in (**a**, **e**, **i**), while the distributions based on a 2D integral equation approach are shown in (**b**, **f**, **j**). The reconstructed SAR distributions based on CSI-EPT are presented in (**c**, **g**, **k**). The relative error between the reconstructed SAR distributions based on CSI-EPT and 3D FDTD is shown in (**d**, **h**, **l**)

The SAR distributions within the non-central slices are depicted in Fig. 6 (slice at $z = + 7.5$ cm) and Fig. 7 (slice at $z = - 2.5$ cm). The reconstructed SAR distribution of the transversal slice at $z = + 7.5$ cm, where $\left| {E_{z} } \right|$ is the dominant field, is in good agreement with the SAR distributions calculated by 3D and 2D forward modeling as shown in the 1st and 2nd column of Fig. 6, respectively. In the fourth column of Fig. 6, the relative error between the reconstructed SAR distributions based on CSI-EPT and 3D FDTD is shown. However, the SAR reconstruction within the slice located at $z = - 2.5$ cm, where the transverse electric field components were not negligible, shows a discrepancy in the central region in a comparison between the 1st and 3rd column of Fig. 7. The discrepancy is due to the fact that transverse electric fields are not considered in a 2D approach, and discrepancies in reconstructed SAR may therefore appear in regions where these transverse fields are not negligible. However, comparison of the 1st and 3rd column of Fig. 7 still shows a good agreement outside the central region as confirmed by the relative error shown in the fourth column of Fig. 7.

## Discussion

Hot spots are a potential risk of high field clinical MRI; prediction of SAR distribution may help to reduce this hazard and is thus essential for MRI quality assurance and patient safety. In this paper, we have exploited the CSI-EPT method to reconstruct electric field and tissue properties and investigated the performance to reconstruct SAR distributions based on $B_{1}^{ + }$ information only. This method takes the integral representations for the electromagnetic field as a starting point, and the electric field and tissue parameters are obtained by iteratively minimizing an objective function which measures the discrepancy between measured and modeled data and the discrepancy in satisfying a consistency equation known as the object equation.

Numerical results illustrate that SAR distributions can be reconstructed based on $B_{1}^{ + }$ information using a 2D implementation of CSI-EPT. In general, a good performance was observed for slices where the transverse components of the electric field were negligible. These results clearly illustrate the ability of CSI-EPT to reconstruct SAR distributions within slices where $E_{z}$ is the dominant field component, which is in general the case for the midplane slice of an RF body coil model [31]. Our studies indicate, however, that a two-dimensional field approximation may also be applied for off-central transverse slices (see Fig. 6). In such cases, a 2D implementation of CSI-EPT would yield reliable SAR reconstruction as well. Unfortunately, it is not a priori known on which off-central slices, the transverse components of the E-field are negligible and we therefore restrict ourselves to the midplane slice when we use a 2D implementation of CSI-EPT. Despite this restriction, the current 2D implementation of CSI-EPT seems to be a promising tool to improve current SAR assessment, since a good agreement was observed between reconstructed SAR distributions and 3D FDTD-based SAR distributions. As can be seen from Figs. 5, 6, and 7, the reconstructed voxel-based, 10 and 1 g SAR distributions show a good overall agreement. To quantify the error in all three cases, we have computed the relative error between the two-dimensional reconstructed SAR based on CSI-EPT (third column in Figs. 5, 6, and 7) and the true SAR distribution as determined by the full 3D FDTD model (first column in Figs. 5, 6, and 7). We observe that the error is small throughout the slice except in some highly isolated regions. These error regions occur mainly because the size of the hot spots is not precisely predicted by our 2D model. Our model does indicate, however, where hot spots can be expected and gives a good overall qualitative indication of the SAR distribution within the slices of interest. Moreover, CSI-EPT is applicable at all fields strength and is not limited to the demonstrated performance at 3T.

In its present form, the CSI-EPT algorithm takes perturbed $B_{1}^{ + }$ field as input and effects due to noise are suppressed by incorporating multiplicative total variation regularization into the CSI-EPT algorithm (see [2]). Additional uncertainties in the $B_{1}^{ + }$ phase may also be taken into account [2]. In practice, measurements of the $B_{1}^{ + }$ phase are based on assumptions regarding the object and coil geometry [17, 32] and this transceive phase assumption can be considered as an uncertainty in the $B_{1}^{ + }$ phase as well. These uncertainties can be taken into account in CSI-EPT by modifying the objective function in a similar manner as in [26]. However, in a number of recent studies [18, 19, 24, 37], the transceive phase assumption is avoided by using multiple independent transmit/receive channels. This opens up possibilities for EPT reconstruction and local SAR estimation [18, 37, 38] free of assumptions regarding the $B_{1}^{ + }$ phase. Although we have presented reconstruction results for a quadrature coil configuration only, CSI-EPT is actually suitable for various antenna settings and can therefore benefit from multiple independent transmit/receive systems as exploited in [18, 37, 38] for assumption-free phase data.

The applicability of the EPT method to electric properties mapping has recently been confirmed in a series of phantom and in vivo experiments with MRI systems [17, 20, 22, 34]. Present work is therefore focused on extending the current implementation of CSI-EPT toward a practical MRI setting using both 2D and 3D field models. Three-dimensional models obviously do not suffer from a restriction to the midplane of the body coil and will provide more accurate reconstruction results in regions where two-dimensional field approximations fail. On the other hand, computation times in 3D will be significantly larger than in 2D due to an increase in the number of unknowns and the application of 3D FFTs. If possible, it is therefore beneficial to use 2D CSI-EPT, which may even provide online SAR reconstructions in the midplane of a body coil.

## Conclusion

Whether a two- or three-dimensional CSI-EPT method is applied, the CSI-EPT method reconstructs, besides the electric properties, also the electric field at no additional computational costs. Given the promising results presented in this paper, we believe that CSI-EPT may prove an important tool toward MR-based SAR reconstruction. In future work, we will therefore focus on developing an efficient implementation of 3D CSI-EPT that allows for complete local SAR assessment inside and outside the midplane of the RF transmit coil.
